# Increased Autophagy-Related 5 Gene Expression Is Associated with Collagen Expression in the Airways of Refractory Asthmatics

**DOI:** 10.3389/fimmu.2017.00355

**Published:** 2017-03-29

**Authors:** Audrey H. Poon, David F. Choy, Fazila Chouiali, Rakhee K. Ramakrishnan, Bassam Mahboub, Severine Audusseau, Andrea Mogas, Jeffrey M. Harris, Joseph R. Arron, Catherine Laprise, Qutayba Hamid

**Affiliations:** ^1^Meakins-Christie Laboratories, Faculty of Medicine, McGill University, Montreal, QC, Canada; ^2^Biomarker Discovery – OMNI, Genentech Inc., South San Francisco, CA, USA; ^3^College of Medicine, University of Sharjah, Sharjah, United Arab Emirates; ^4^OMNI Early Clinical Development, Genentech Inc., South San Francisco, CA, USA; ^5^Immunology Discovery, Genentech Inc., South San Francisco, CA, USA; ^6^Department of Sciences Fondamentales, Université du Québec à Chicoutimi, Chicoutimi, QC, Canada

**Keywords:** autophagy, ATG5, asthma, collagen, airway remodeling

## Abstract

**Background:**

Fibrosis, particularly excessive collagen deposition, presents a challenge for treating asthmatic individuals. At present, no drugs can remove or reduce excessive collagen in asthmatic airways. Hence, the identification of pathways involved in collagen deposition would help to generate therapeutic targets to interfere with the airway remodeling process. Autophagy, a cellular degradation process, has been shown to be dysregulated in various fibrotic diseases, and genetic association studies in independent human populations have identified autophagy-related 5 (ATG5) to be associated with asthma pathogenesis. Hence, the dysregulation of autophagy may contribute to fibrosis in asthmatic airways.

**Objective:**

This study aimed to determine if (1) collagen deposition in asthmatic airways is associated with ATG5 expression and (2) ATG5 protein expression is associated with asthma *per se* and severity.

**Methods:**

Gene expression of transforming growth factor beta 1, various asthma-related collagen types [collagen, type I, alpha 1; collagen, type II, alpha 1; collagen, type III, alpha 1; collagen, type V, alpha 1 (COL5A1) and collagen, type V, alpha 2], and ATG5 were measured using mRNA isolated from bronchial biopsies of refractory asthmatic subjects and assessed for pairwise associations. Protein expression of ATG5 in the airways was measured and associations were assessed for asthma *per se*, severity, and lung function.

**Main results:**

In refractory asthmatic individuals, gene expression of ATG5 was positively associated with COL5A1 in the airways. No association was detected between ATG5 protein expression and asthma *per se*, severity, and lung function.

**Conclusion and clinical relevance:**

Positive correlation between the gene expression patterns of ATG5 and COL5A1 suggests that dysregulated autophagy may contribute to subepithelial fibrosis in the airways of refractory asthmatic individuals. This finding highlights the therapeutic potential of ATG5 in ameliorating airway remodeling in the difficult-to-treat refractory asthmatic individuals.

## Introduction

The Global Initiative for Asthma estimated that globally there are 300 million people who suffer from asthma and the number is expected to reach 400 million by 2025 (1). Among asthmatic individuals, between 5 and 10% are considered severe who need a combination of oral steroids, inhaled steroids, short-acting bronchodilator, long-acting bronchodilator, and leukotriene modifiers to control their asthma. However, a proportion of these severe asthmatic subjects, despite aggressive treatment schemes continue to have exacerbations, obstructive airways, emergency visits, and even near fatal asthma attacks. The injury and repair associated with severe persistent asthma results in irreversible airflow obstruction due to airway remodeling (2, 3). Fibrosis is an important characteristic of tissue remodeling, and in asthmatic airways, fibrosis is associated with increased collagen deposition in the subepithelium (4–7). Simplistically put, in normal airways, collagen and other extracellular matrix (ECM) proteins are deposited and degraded in a homeostatic fashion; yet in asthmatic airways, such homeostasis is dysregulated, as reviewed elsewhere (8). In asthmatic airways, collagen type I (4), type III (4, 9), and type V (9, 10), among other ECM proteins, were found in greater quantity than in non-asthmatic airways. Furthermore, the presence of fibrosis below the epithelium of airways is associated with asthma severity and lung function decline (6). While commonly used asthma medications are effective in reducing inflammation and dilating constricted airways, they are ineffective in reducing or preventing fibrosis (6, 11, 12).

Autophagy is a cellular degradation process in which the cell hydrolytically removes cytoplasmic contents, such as damaged organelles and protein aggregates, by first engulfing the target within a double membrane vacuole and followed by fusion with lysosomes, as reviewed elsewhere (13, 14). During autophagy, autophagy-related 5 (ATG5) is covalently conjugated with ATG12 and interacts with ATG16 to form the ATG12–ATG5–ATG16 complex (15). This complex enhances the formation of the membrane destined to form an autophagosome and is thus, vital for autophagosome formation (16). Intricate relationships exist between autophagy and other forms of cell death (i.e., apoptosis and necrosis) (17, 18). It has been postulated that autophagy serves as a cell survival mechanism to remove triggers that are threatening cell survival, yet when such threats become overwhelming, cell death processes such as apoptosis and necrosis take over (18, 19). Dysregulation of autophagy has been linked to fibrosis in a number of fibrotic diseases, including cirrhosis (20), idiopathic pulmonary fibrosis (21), and renal fibrosis (22). The upregulation of autophagy during activation of fibrogenic cells, such as hepatic stellate cells from mice as well as hepatitis B-, hepatitis C-virus-infected human liver, and human fibroblasts from idiopathic pulmonary fibrosis, suggests that autophagy is a central pathway in fibrosis (23, 24). The loss of autophagy function, with specific autophagy inhibition by siRNAs against *Atg5*, results in the attenuation of matrix accumulation and fibrogenesis in stellate cells and renal, embryonic and lung fibroblasts (23), further supporting the role of autophagy in the fibrotic process. Recently, elevated autophagic activities have been detected in cells from sputum and blood from severe asthmatic patients as compared to the milder asthmatics and healthy controls (25). Furthermore, two candidate gene association studies detected associations between variations in the gene encoding ATG5 and asthma (26, 27), and elevated ATG5 gene expression was found in the nasal epithelium of children with acute asthma as compared to those with no asthma or stable asthma (27). In addition to asthma *per se, ATG5* polymorphism was associated with lung function in asthmatic individuals (26). This genetic association coupled with the histological observation of increased autophagosomes in moderately severe asthmatics provides evidence of autophagy in the pathogenesis of asthma (25, 26). Stemming from these reports, we hypothesize that ATG5 expression is associated with collagen deposition in severe asthmatic patients.

## Materials and Methods

### Sample Collections

Gene expression measurement using microarray was performed using RNA isolated from bronchial biopsy samples of study participants in the Bronchoscopic Exploratory Research Study of Biomarkers in Corticosteroid-refractory Asthma (BOBCAT) study (28). The BOBCAT study was a multicenter study conducted in Canada, United States, and United Kingdom, and patient recruitment has been described previously (28). Briefly, patients with uncontrolled moderate-to-severe asthma accompanied by forced expiratory volume in one second (FEV_1_) percent (%) predicted of 40–80%, airway obstruction of >12% and reversibility with a short-acting bronchodilator or methacholine sensitivity (PC_20_) <8 mg/ml in the past 5 years were recruited. The asthma of the participants must be refractory as defined by at least two exacerbations in the previous year or an asthma control quality (ACQ) score of >1.50 while on high-dose inhaled corticosteroid (ICS) (>1,000 μg of fluticasone or equivalent daily) with or without long-acting β-agonist. Processing of the bronchial biopsy tissues for RNA isolation and gene expression microarray analyses has been described previously (29).

Protein expression was measured in bronchial biopsy tissues obtained from fiberoptic bronchoscopy of asthmatic and non-asthmatic healthy subjects archived at the Tissue Bank of the Respiratory Health Network of the Fonds de Recherche du Québec – Santé (McGill University Health Centre site). Patient recruitment and sample processing have been described previously (6, 30). Asthma severity (mild, moderate, and severe) was determined based on medication usage, frequency of exacerbation, and lung function as previously described (30, 31). Briefly, severe asthma subjects met the criteria proposed by the American Thoracic Society workshop on refractory asthma (32). Moderate asthmatic subjects were individuals with persistent asthma whose symptoms were under-control with a dosage between 176 and 800 μg/d of fluticasone (or equivalent) with or without add-on controller medication, no more than two steroid bursts in the past 12 months and none in the past 3 months with total days on oral steroids <30 days in the prior 12 months, predicted FEV_1_ >70% and >90% of personal best from the past 2 years, and a maximum of one unscheduled visit for asthma in the prior 12 months. Mild asthmatic subjects were individuals with prebronchodilator predicted FEV_1_ >80% and treated with either no or low-to-moderate dose of ICS (<880 μg fluticasone or equivalent). In addition to asthmatic subjects, non-asthmatic subjects with no history of asthma diagnosis, predicted FEV_1_ >90% and free of respiratory or systemic diseases, were included as control subjects.

All subjects have given their informed consent in accordance with the Declaration of Helsinki, and the study has been approved by the Research Ethics Board of the Research Institute-McGill University Health Centre. The BOBCAT protocol was approved by the Copernicus Group independent review board and respective institutional review boards associated with other participating study centers in the United States, Canada, and UK.

### Gene Expression

Gene expression data were available from previously performed microarray analyses (33). Briefly, amplified single-stranded cDNA from homogenized bronchial biopsy tissues was hybridized to Affymetrix (Santa Clara, CA, USA) U133 plus 2.0 arrays, and array images were analyzed with Affymetrix GeneChip Expression Analysis Software. Gene expression data in the airways of various asthma-related collagen types [collagen, type I, alpha 1 (COL1A1); collagen, type II, alpha 1 (COL2A1); collagen, type III, alpha 1 (COL3A1); collagen, type V, alpha 1 (COL5A1) and collagen, type V, alpha 2 (COL5A2)], transforming growth factor beta 1 (TGFB1), and ATG5 were obtained.

### Immunocytochemistry

Immunocytochemistry staining of formalin-fixed paraffin-embedded biopsy samples was performed to determine the protein level of ATG5. ATG5 immunoreactivity was detected using an ATG5 specific antibody (Abcam, Cambridge, MA, USA, ab109490) on 5 µm thick tissues as previously described (34). Briefly, heat-activated antigen-retrieval in citrate buffer was performed to expose antigens; endogenous peroxidase activity was blocked with 1% H_2_O_2_, protein detection and signal amplification were achieved with streptavidin-horseradish peroxidase complex (Dako, Carpinteria, CA, USA), brown color stains were developed by 3,3′-diaminobenzidine (Dako, Carpinteria, CA, USA), and tissues were counter stained with hematoxylin and lithium carbonate. Image analyses were performed using the image processing program, ImageJ (version 1.46). Protein expression of ATG5 in the submucosal area was measured as the proportion of positively stained area.

### Statistical Analyses

Microarray data analyses were performed using Bioconductor in the R statistical environment as previously described (35). Pairwise correlation of gene expressions was performed using Spearman’s rank order correlation. A Bonferroni corrected *p* value <0.004 was considered as statistically significant. The association between ATG5 protein expression in the bronchial biopsy samples and asthma *per se*, severity, and lung function were assessed by Wilcoxon, Wilcoxon rank sums, and Pearson’s correlation tests, respectively.

## Results

### Population Characteristics

Bronchial biopsy tissues from 35 refractory asthmatic subjects that participated in the BOBCAT study were used to assess for pairwise correlations between autophagy and different collagen subtypes gene expressions. Patients characteristics have previously been published (28). Briefly, the mean age of the subjects was 46 years (SD = 11) with 62.9% of the subjects being male. The mean FEV_1_% predicted was 61% (SD = 12). The mean ACQ score was 2.6 (SD = 0.8). Bronchial biopsy tissues from 42 asthmatics (15 mild, 12 moderate, and 15 severe) and 15 non-asthmatic healthy subjects were used to measure and assess correlation between ATG5 protein expression and asthma *per se* and severity. The mean ages of the four groups (normal, mild, moderate, and severe) were 32.7 years (SD = 14.9), 31.6 years (SD = 9.5), 42.1 years (SD = 10.6), and 40.9 years (SD = 8.1), respectively. The percentages of the samples being female were 60, 63, 50, and 27%, respectively. In terms of lung function, the mean FEV_1_% predicted values of the four groups were 109.1% (SD = 14.9), 90.0% (SD = 15.6), 90.4% (SD = 14.5), and 58.3% (SD = 15.4), respectively. The mean FEV_1_/forced vital capacity values were 0.83 (SD = 0.07), 0.76 (SD = 0.10), 0.77 (SD = 0.09), and 0.67 (SD = 0.13), respectively.

### Gene Expression of TGFB1 in the Airways Is Associated with COL1A1 Gene Expression in Refractory Asthmatic Subjects

Pairwise gene expression comparisons demonstrated a significant correlation between *TGFB1* and *COL1A1* (ρ = 0.59, *p*-value = 2.4 × 10^−4^) (Table 1). The correlation between *TGFB1* and *COL1A2* demonstrated a positive trend but was not significant after correction (ρ = 0.32, *p*-value = 0.06). No significant correlations or trends were observed between *TGFB1* and the other investigated collagen types in this study [*COL3A1* (ρ = 0.01, *p*-value = 0.95), *COL5A1* (ρ = 0.07, *p*-value = 0.71), and *COL5A2* (ρ = 0.01, *p*-value = 0.96)]. Therefore, *COL1A1* gene expression positively correlated with *TGFB1* gene expression in refractory asthmatics.

**Table 1:** **Pairwise correlations between gene expression of various collagen types and *ATG5* and *TGFB1* in bronchial biopsy tissues of refractory asthmatic individuals**.

	*COL1A1*	*COL1A2*	*COL2A1*	*COL3A1*	*COL5A1*	*COL5A2*	*ATG5*
*TGFB1*	ρ = 0.59, p* = 2.4 × 10^−4^	ρ = 0.32, *p* = 0.06	Did not analyze	ρ = 0.01, *p* = 0.95	ρ = 0.07, *p* = 0.71	ρ = 0.01, *p* = 0.96	ρ = 0.22, *p* = 0.21
*ATG5*	ρ = 0.42, *p* = 0.01	ρ = 0.25, *p* = 0.14	ρ = −0.3, *p* = 0.08	ρ = 0.33, *p* = 0.05	ρ = 0.72, *p* = 2.9 × 10^−6^	ρ = 0.30, *p* = 0.08	

*Bonferroni adjusted *p*-value threshold for statistical significance (i.e., 12 tests) is 0.004.

### Gene Expression of ATG5 in the Airways Is Associated with COL5A1 Gene Expression in Refractory Asthmatic Subjects

Pairwise gene expression comparisons demonstrated a significant correlation between *ATG5* and *COL5A1* (ρ = 0.72, *p*-value = 2.9 × 10^−6^) (Table 1). The correlation between *ATG5* and *COL1A1* demonstrated a positive trend but was not significant after correction (ρ = 0.42, *p*-value = 0.01). No significant correlations or trends were observed between *ATG5* and the other investigated collagen types in this study [COL1A2 (ρ = 0.25, *p*-value = 0.14), COL2A1 (ρ = −0.3, *p*-value = 0.08), COL3A1 (ρ = 0.33, *p*-value = 0.05), and COL5A2 (ρ = 0.30, *p*-value = 0.08)]. Therefore, *COL5A1* gene expression positively correlated with *ATG5* gene expression in refractory asthmatics.

### Protein Expression of ATG5 in the Airways Is Not Associated with Asthma, Asthma Severity, or Lung Function in Asthmatic Subjects

**Figure 1:**
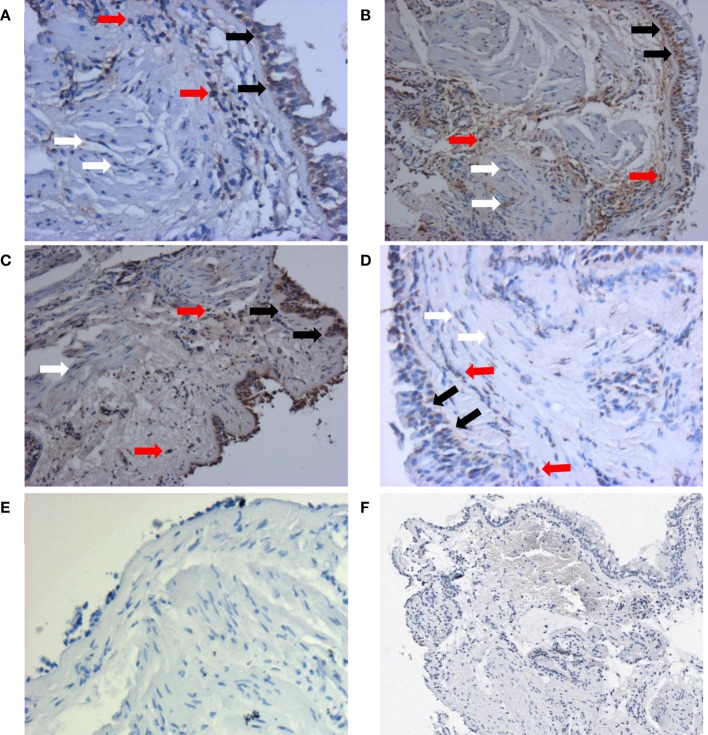
**Representative staining patterns of ATG5 protein expression from each group were shown here: non-asthmatic control (A), mild (B), moderate (C), and severe (D)**. Monoclonal antibody for ATG5 was used as the primary antibody and developed with 3,3′-diaminobenzidine diaminobenzidine (brown). Nuclei were stained with hematoxylin (blue). Negative controls of ATG staining were performed using IgG1 isotype **(E)** and pre-absorption with ATG antigens **(F)**. Pictures were taken at 200× magnification. Positive stainings could be detected in epithelial cells (black arrows), airway smooth muscle cells (white arrows), and inflammatory cells (red arrows). Enlarged staining patterns can be found in Figure S1 in Supplementary Material.

**Figure 2:**
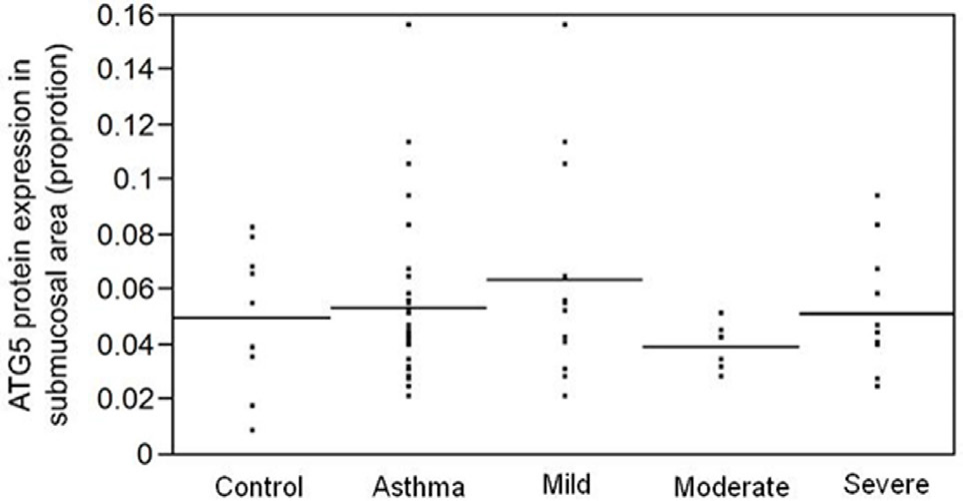
**ATG5 protein expression, measured as proportion of positively stained area in the submucosa, is not associated with asthma *per se* (*p* = 0.1) or with asthma severity (*p* = 0.7)**.

Since fibrosis is often associated with asthma severity and decline in lung function (6), and *ATG5* gene expression demonstrated positive correlation with *COL5A1* gene expression, the ATG5 protein expression in the asthmatic airways was investigated to determine the association, if any, with asthma *per se*, severity, and lung function. ATG5 proteins were detected in the epithelium, airway smooth muscle cell bundles, and inflammatory cells in all asthmatic and non-asthmatic subjects (Figure 1). Pre-absorption of the ATG5 monoclonal antibodies with ATG5 peptides prevented any positive staining of the biopsy tissues (Figure 1F). ATG5 protein expression in the submucosal area was measured and expressed as a proportion of positively stained tissue area. No significant difference was detected in the ATG5 expression between non-asthmatic control and asthmatic subjects (Wilcoxon *p*-value = 0.1) (Figure 2). When asthmatic subjects were stratified by severity (mild, moderate, and severe), no significant difference in ATG5 expression was observed among the three asthmatic severity groups and with the non-asthmatic group (*p*-value = 0.7 Wilcoxon rank sums) (Figure 2). In terms of lung function in asthmatic subjects, ATG5 protein expression did not correlate with FEV_1_% predicted in asthmatic subjects [Spearman’s rank correlation coefficient (ρ) = 0.04, *p* = 0.83] or when stratified by asthma severity (0.3 < Spearman’s ρ < 0.8, 0.26 < *p* < 0.83) (data not shown).

## Discussion

Albeit the genetic findings of association between ATG5 polymorphism and various asthma-related traits in a number of independent populations, no association was found between the ATG5 protein level and either the presence of asthma *per se*, severity of asthma, or by lung function in this study. A number of factors may contribute to the lack of association at the protein level despite associations detected at the gene level. For example, genotypes of the bronchial biopsy tissues were not determined due to limited availability of archived tissues. Furthermore, elevated gene expression was observed in nasal mucosal cells of acute asthmatic children as compared to stable asthmatic and control children (27). The biological consequence may only be detectable in the downstream autophagic pathway. The detection of LC3B-II punctae using immunocytochemistry is often used to indicate the presence of autophagy (24); however, the use of archived formalin-fixed paraffin-embedded tissues in this study limited its use. In this study, the investigation was focused on ATG5 proteins in the submucosal area of the airways; hence, the impact of the genetic and gene expression association may not be carried to protein expression in different cell types. Finally, the phenotypes of interest were different from previous studies reporting genetic associations. Although the disease of investigation was asthma, the underlying mechanism for the genetic association with acute asthma in children or with FEV_1_% predicted in asthmatic subjects may, and likely be, different from that of severity.

Subepithelial fibrosis is a hallmark of asthmatic airways, and fibrosis can be primarily attributed to the deposition of collagen of types I, III, and V as well as fibronectin (6). The findings of positive association between the gene expression of various collagens (i.e., COL5A1 and less significantly COL1A1) and ATG5 supported the speculation that enhanced autophagy is associated with asthma pathogenesis and in particular collagen deposition. It has been suggested that TGFB1 is a pro-fibrotic element present in asthmatic tissues (6, 36), as evidenced by the gene expression data of the BOBCAT study. TGFB1 gene expression positively correlated with type 1 collagen gene expression in this study. This is in concordance with the observation that TGFB1 simultaneously promotes COL1A2 synthesis and autophagy induction in human atrial myofibroblasts, and ATG5 knockout of mouse embryonic fibroblasts is associated with a parallel decline in the fibrotic effect of TGFB1 when compared to wild-type cells, further stressing the role of autophagy in TGFB1-induced fibrosis (37). Interestingly, in this study TGFB1 gene expression did not correlate with COL5A1 and COL5A2, yet ATG5 gene expression correlated with the type V collagen gene expression. Type V collagen is a minor collagen that is intercalated within fibrils of the major lung collagen, type I collagen (38). Under normal conditions, the epitopes of the type V collagen are masked within the fibrils; yet in conditions with prominent tissue remodeling, the type V epitopes are exposed and have been shown to induce autoimmunity in a murine model of allergic airway disease (10), lung transplant-associated obliterative bronchiolitis (39), and idiopathic pulmonary fibrosis (40). In the context of asthma, an observational study of asthmatic subjects has detected higher levels of type V collagen antibody in the serum of asthmatic subjects than in non-asthmatic healthy subjects (10). Histopathological examination of a lung biopsy of an individual with fatal asthma also demonstrated greater type V collagen staining than normal lung biopsy (10). The murine model of allergic airway disease further demonstrated the positive association between anti-type V collagen antibody and IgE antibody production, and the protective effect of type V collagen-induced tolerance in airway resistance and airway hyperresponsiveness (10). Given that the BOBCAT study subjects are of moderate-to-severe asthma severity, airway remodeling is likely to be prevalent, and increased synthesis and deposition of type V collagen may be an important contributor to the associated fibrosis in these asthmatics.

Fibrosis in different organs has been associated with both autophagic upregulation as well as downregulation, emphasizing the diversity in the functional role of autophagy in tissue repair (41). On the one hand, it has been shown that in proximal epithelial cells, ATG5-mediated autophagy reduced type I collagen deposition by blocking the G_2_/M phase arrest (42), a cell cycle phase whose arrest would initiate DNA repair and synthesis of pro-fibrotic factors (43). Furthermore, bleomycin-induced pulmonary fibrosis in a mouse model led to increased autophagy activation in the lungs as revealed by upregulated ATG5 protein expression levels and increased autophagosome formation (44). However, deficient autophagy in this model enhanced lung fibrosis, which was characterized by upregulation of collagens, COL1A2 and COL3A1. On the other hand, in human oral fibroblasts, suppression of autophagy led to reduction in type I collagen (i.e., COL1A2) gene expression, promotion of apoptosis, and suppression of proliferation (45). Additionally, prolonged starvation of human embryonic lung fibroblasts triggered the simultaneous activation of myofibroblast differentiation, which was accompanied by increased COL1A1 and COL3A1 at mRNA or protein levels, and autophagy (41). Autophagy inhibition was shown to prevent collagen mRNA and protein levels and myofibroblast differentiation (41). These discrepancies clearly demonstrate tissue and cell specificity in the downstream effects of autophagy. Other models of fibrosis suggested that autophagy may regulate fibrosis through a large number of pathways including the activation of the unfolded protein response (46), the activation of the IL-17A/STAT3 signaling pathway (47), the suppression of mitochondrial reactive oxidative species–NF-κB-IL1α/β pathways (48), and the degradation of activated caspase-8 (49).

The mechanism behind the observed association between autophagy and type V collagen production is unknown and elusive. Angiotensin may be involved in tissue remodeling, and angiotensin II type I receptor signaling has been shown to induce autophagy in cardiomyocytes (50). Angiotensin II stimulation was observed to activate autophagy in rat cardiac fibroblasts both *in vitro* and *in vivo*, and ATG5 knockdown augmented angiotensin II-mediated accumulation of collagen type I (51). In another study, angiotensin II type I receptor antagonist, valsartan, suppressed types III and V collagen synthesis by modulating TGFB1 expression at the mRNA and protein levels (52), suggesting a plausible role of autophagy in type V collagen deposition *via* angiotensin II type I receptor signaling. In order to draw a mechanistic conclusion of the ATG5–type V collagen association in this study, further investigations involving various cell types such as fibroblasts, epithelial cells, and smooth muscle cells need to be studied separately. However, the findings that type V collagen and autophagy are associated in the lung tissues of moderate-to-severe asthmatic subjects are novel and exciting. Though there have been recent health authority approvals of two drugs to treat idiopathic pulmonary fibrosis, no pharmaceutical agents have yet been shown to directly ameliorate or reverse fibrosis. This finding supports ATG5 as a new target for anti-fibrotic drug development.

## Author Contributions

AP, DC, SA, AM, JH, JA, CL, and QH conceptualized and designed the study; acquired, analyzed, and interpreted the data generated; drafted, revised, and approved the manuscript; and agreed to be accountable for all aspects of the work. RR and BM edited, revised, and analyzed the data and content in the manuscript.

## Conflict of Interest Statement

DC is employed by, has received patents from, and has stock options in Genentech. QH has received research support from Meakins-Christie Laboratories. JH is employed by and has stock options in Genentech. JA is employed by Genentech, has received payment for lectures from the American Asthma Association, has patents with Genentech, and has stock in Roche Holdings. The rest of the authors declare that they have no relevant conflicts of interest.

## Supplementary Material

Figure S1 **ATG5 positive staining in various cell types in a bronchial biopsy tissue of a severe asthmatic subject**. Nuclei were stained with hematoxylin (blue). Positive stainings could be detected in epithelial cells (black arrow), airway smooth muscle cells (white arrow), and inflammatory cells (red arrow).
