# Supplementary figures and images for: Increased Autophagy-Related 5 Gene Expression Is Associated with Collagen Expression in the Airways of Refractory Asthmatics
Source: Front Immunol. 2017 Mar 29;8:355. doi: 10.3389/fimmu.2017.00355 (PMC5372794; doi:10.3389/fimmu.2017.00355)

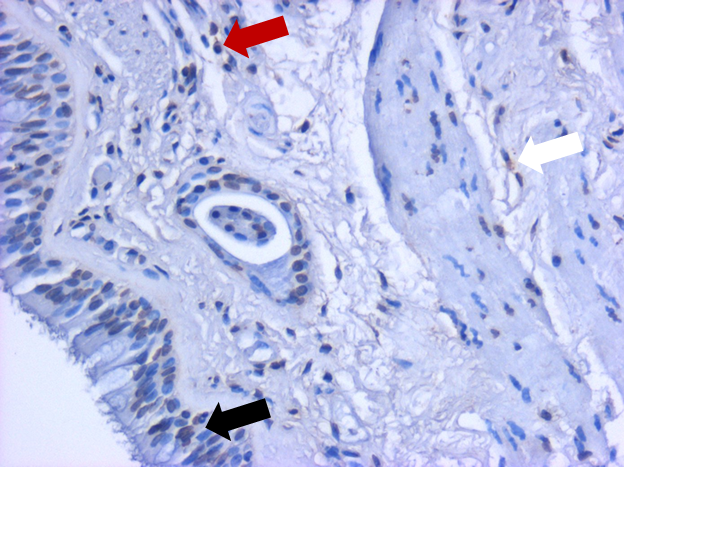

Supplement: Figure S1 — ATG5 positive staining in various cell types in a bronchial biopsy tissue of a severe asthmatic subject. Nuclei were stained with hematoxylin (blue). Positive stainings could be detected in epithelial cells (black arrow), airway smooth muscle cells (white arrow), and inflammatory cells (red arrow). [file Image_1.TIF]
